# Strontium ranelate inhibits wear particle-induced aseptic loosening in mice

**DOI:** 10.1590/1414-431X20187414

**Published:** 2018-07-10

**Authors:** Tianxiang Geng, Shouxuan Sun, Haochen Yu, Haohui Guo, Mengxue Zheng, Shuai Zhang, Xi Chen, Qunhua Jin

**Affiliations:** 1 Ningxia Medical University, General Hospital of Ningxia Medical University, Yinchuan, Ningxia, China; 2 Department of Orthopedic Surgery, General Hospital of Ningxia Medical University, Ningxia Medical University, Yinchuan, Ningxia, China

**Keywords:** Strontium ranelate, Aseptic loosening, Sclerostin, Bone remodeling, Canonical Wnt pathway

## Abstract

The imbalance between bone formation and osteolysis plays a key role in the pathogenesis of aseptic loosening. Strontium ranelate (SR) can promote bone formation and inhibit osteolysis. The aim of this study was to explore the role and mechanism of SR in aseptic loosening induced by wear particles. Twenty wild-type (WT) female C57BL/6j mice and 20 sclerostin-/- female C57BL/6j mice were used in this study. Mice were randomly divided into four groups: WT control group, WT SR group, knockout (KO) control group, and KO SR group. We found that SR enhanced the secretion of osteocalcin (0.72±0.007 in WT control group, 0.98±0.010 in WT SR group, P=0.000), Runx2 (0.34±0.005 in WT control group, 0.47±0.010 in WT SR group, P=0.000), β-catenin (1.04±0.05 in WT control group, 1.22±0.02 in WT SR group, P=0.000), and osteoprotegerin (OPG) (0.59±0.03 in WT control group, 0.90±0.02 in WT SR group, P=0.000). SR significantly decreased the level of receptor activator for nuclear factor-κB ligand (RANKL) (1.78±0.08 in WT control group, 1.37±0.06 in WT SR group, P=0.000) and improved the protein ratio of OPG/RANKL, but these effects were not observed in sclerostin-/- mice. Our findings demonstrated that SR enhanced bone formation and inhibited bone resorption in a wear particle-mediated osteolysis model in wild-type mice, and this effect relied mainly on the down-regulation of sclerostin levels to ameliorate the inhibition of the canonical Wnt pathway.

## Introduction

Total joint replacement is one of the most effective treatments for end-stage arthritis and is widely used in clinical treatment (1). Aseptic loosening is among the long-term complications of total joint replacement and an important factor affecting the success rate. The pathogenesis of aseptic loosening is not clear, but previous studies have indicated that an imbalance in osteogenesis and osteolysis around the prosthesis is the root cause of aseptic loosening (2,3). The canonical Wnt signaling pathway, which exists in a variety of tissues and cells, is an important signaling pathway affecting osteoblast function. The pathway is composed of frizzled protein, lipoprotein receptor-related protein (LRP), and transcriptional regulator β-catenin protein (4). Activation of the signal pathway can promote the differentiation and stimulate the proliferation of osteoblasts, leading to enhanced bone metabolism (5,6).

Experiments have shown that knockout of the *Lrp5* gene in mice led to reduced osteogenesis (7,8). The canonical Wnt pathway does not directly affect osteoclasts, but rather regulates the secretion of osteoprotegerin (OPG). OPG is secreted by various cells such as osteoblasts and mesenchymal stem cells, and is a soluble competitive decoy receptor for receptor activator for nuclear factor-κB (RANK), can inhibit the NF-κB signaling pathway by decreasing RANKL-RANK binding. Thus, OPG inhibits osteoclast differentiation and activation, and induces apoptosis.

Blocking the canonical Wnt pathway can inhibit the proliferation of osteoblasts and promote osteoblast apoptosis, leading to reduced bone formation. Secretion of OPG in osteoblasts is decreased, decreasing the OPG/RANKL ratio and eventually leading to increased osteoclast production (9–11). The canonical Wnt pathway can be regulated by a variety of protein families, including sclerostin (SOST), Dickkopf-related protein, and secreted frizzled-related proteins (12). SOST, a protein secreted by bone cells, binds to the canonical Wnt pathway receptor LRP on the osteoblast membrane to inhibit signal pathway activity and has a negative regulatory effect on bone formation (13). SOST gene deletion can cause sclerostenosis. Previous studies showed that there is no significant change in body weight between SOST knockout mice and wild-type mice, but the lumbar L3 bone mineral density and bone volume (BV) were significantly higher than those in wild-type mice, a decrease in bone resorption was observed in the distal femur of aged mice osteoclasts, and the number of osteoclasts in females was slightly lower than that in wild-type mice. In SOST gene knockout mice, LRP expression was not affected (7). An *in vivo* experiment has shown that SOST antibody can increase bone formation, bone mass, and bone strength in open osteotomy rat models (14).

Local inflammation plays an important role in the pathogenesis of aseptic loosening. Experiments have shown that wear particles stimulate macrophages to release a variety of inflammatory factors, such as interleukin (IL)-1β and tumor necrosis factor (TNF)-α (15,16). These inflammatory factors can promote the proliferation and differentiation of osteoclasts through a variety of signaling pathways and ultimately promote bone resorption around the prosthesis (17,18). The interface membranes are pathological tissues around the failed prosthesis that contain high levels of IL-1β and TNF-α (19).

Strontium ranelate (SR), which has been demonstrated as an effective anti-osteoporosis remedy, has the potential to reduce the incidence of spinal and hip fracture in postmenopausal women (20). SR has been shown to promote the proliferation of pre-osteoblastic cells, suppress osteoclast differentiation and activity, and increase osteoclast apoptosis (21).

In this study, a mouse model that simulates artificial joint replacement and reflects the interaction between wear particles and periprosthetic tissue (22) was used to investigate the possibility of SR treatment by examining the underlying molecular and biomechanical mechanisms, and if it is a possible candidate to reduce the incidence of this confounding complication.

## Material and Methods

### Wear particle preparation

Unmixed titanium particles (Ti-6Al-4V, Zimmer Company, USA) with an average particle diameter of 5 μm were used. Prior to injection, the granules were rinsed in 70% ethanol at room temperature for 48 h and autoclaved at 180°C for 6 h to remove endotoxins. The wear particles were tested for endotoxin using a commercial test kit (E-Toxate; Sigma, USA) (23).

### Animals

In this study, twenty 8-week-old wild-type female C57BL/6j mice and twenty 8-week-old SOST^-/-^ female C57BL/6j mice were used (weight: 26±2 g). SOST^-/-^ mice were purchased from Shanghai Genechem Co., Ltd. (China). All mice were housed in mechanically ventilated cages (4–5 mice per cage) and kept at constant temperature (25°C) and pressure and 12 h/12 h light/dark cycle with free access to water and food. The experimental protocol was in accordance with the NIH guidelines for experimental animals and was approved by the Ethics Committee of Ningxia Medical University General Hospital.

### Experimental groups and treatments

The wild-type mice were randomly divided into the WT control group, WT strontium ranelate (SR) group, and the SOST-/- mice (KO) were randomly divided into KO control group and KO SR group. The randomization was repeated until 40 mice were divided into 4 groups, 10 mice per group. All mice received a rat model of lower limb prosthesis. The experimental method is described elsewhere (22). Intraperitoneal injection (0.6% pentobarbital sodium) was used to induce general anesthesia, and then all mice were treated with the rat joint prosthesis model in the right lower limb. In a sterile environment, the tibial plateau was exposed using the medial platform method and a titanium pin was gently implanted in the proximal tibia so that the surface of the pin and tibial plateau remained in the same plane. Titanium-alloy pins (TA1) of 0.8 mm diameter and 5 mm long with a flat large top of 1.4 mm diameter were used. Sections were washed with physiological saline containing 100 U/mL penicillin and 100 mg/mL streptomycin, each of which was sutured with absorbable sutures. The tibial pelvis of the mouse was injected with 10 μL of titanium suspension (4 × 10^4^ titanium particles in biological saline) before a titanium screw was inserted. Twenty microliters of granules were injected into the capsule every 2 weeks and continued until the end of the experiment. After 1 week of adaptive feeding, the WT SR group and the KO SR group were given SR (S12911–2, PROTELOS^¯^, France) at 625 mg·kg^-1^·d^-1^ for 7 days. The WT control group and the KO control group were given the same dose of physiological saline. Animals were sacrificed 12 weeks after treatment by carbon dioxide asphyxiation.

### Ti prosthesis steadiness examined by pullout test

After sacrifice, the tibia containing the titanium nail was removed from the body. To expose the head of the Ti implant, all muscles and tissues around the bone were carefully removed. Each bone was fixed to a special fixture using dental cement to align the long axis of the implant with the long axis of the HP-100 controlled electronic universal testing machine (Leqing Zhejiang Instrument Scientific Co., Ltd., China). While controlling the position of the mouse limbs and custom fixtures, the HP-100 pulled the stitches from the tibia at a speed of 2.0 mm/min. The load data was registered by an automated software (Edburg, Yueqing Instrument Co., Ltd, China).

### Micro-CT (µCT) scanning

After removing all soft tissue, the tibia from 4 mice in each group was fixed in 4% paraformaldehyde. The fixed bones were scanned by µCT (SkyScan 1176; SkyScan, Kontich, Belgium) at a resolution of 9 μm, exposure time of 900 ms, power of 45 kW, and current of 550 mA. According to CT analysis, the BV fraction (BV/total volume (TV), trabecular thickness (TbTh), trabecular number (TbN), BV, trabecular separation distribution (TbSp), and specific bone surface (BS/BV) around the tibia of the Ti nail were calculated and reconstructed following acquisition using automatic data analysis software (NRecon, Skyscan, Brucker microCT, Belgium).

### Enzyme-linked immunosorbent assay (ELISA)

ELISA was performed using mouse sera to detect interleukin (IL)-1β and TNF-α protein levels. Quantitative analysis was performed using mouse-specific ELISA kits (TNF-α and IL-lβ, NeoBioscience, China).

### Western blotting

The tissue surrounding the implant was frozen in liquid nitrogen and then ground with a chilled mortar and pestle. RIPA buffer containing 1 mM PMSF (KeyGEN Biotech, China) was used to lyse the tissue and release whole protein. Protein concentration was measured by using a BCA assay kit (KeyGEN Biotech). Next, 30 μg of protein mixed with 5X loading buffer was separated by 12% Tris-glycine SDS-PAGE and then transferred to polyvinylidene fluoride membranes (Millipore, USA). The membranes were incubated in TBST (Tris-buffered saline, 0.5% Tween-20) containing 5% nonfat dry milk for 1 h at room temperature and incubated with different primary antibodies (OCN, 1:1000, Abcam, UK; Runx2, 1:1000, Abcam; OPG, 1:1000, Abcam; RANKL 1:1000, Abcam; β-catenin, 1:1000, Abcam; SOST, 1:1000, Abcam; β-actin, 1:1000, Cell Signaling Technology, Danvers, USA; GAPDH, Cell Signaling Technology, USA) overnight at 4°C. The membranes were washed with TBST 3 times and incubated with the secondary antibodies (1:5000, ZSGB-BIO, China) for 1 h at room temperature. ECL western-blotting detection reagent (KeyGEN Biotech) was used to test the bands. Quantity One Software (Bio-Rad, Hercules, USA) was used for semi-quantitative analysis.

### Statistical analysis

Data are reported as the means±SD. Results were analyzed by one-way analysis of variance for differences among the four groups. The least significant differences *post hoc* test was performed to detect the means between different groups. P values ≤0.05 were considered significant. SPSS 19.0 (SPSS, Inc., USA) was used for analysis.

## Results

### Treatment with SR improved the contact state between the prosthesis and the bone of mice

**Figure 1.**
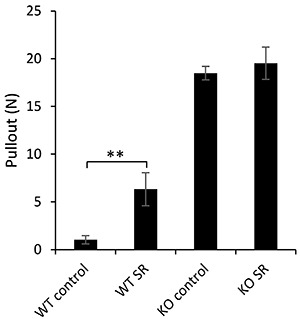
Pullout force results to estimate bone bonding capacity to Ti pin implantations. Data are reported as means±SD. **P<0.01 (ANOVA). SR: strontium ranelate; WT: wild type; KO: knockout.

The special clamp was sufficiently powerful to hold the titanium nail during the pullout test. The average load of pulling was 1.03±0.44 N for the WT control group and 18.49±0.71 N for the knockout (KO) control group. There were significant differences in pulling force between the WT control group and WT SR group (6.32±1.73 N, P=0.000). Compared to the KO control group, the average pulling load of the KO SR group (19.53±1.70 N, P=0.349) was not significantly different (Figure 1).

**Figure 2.**
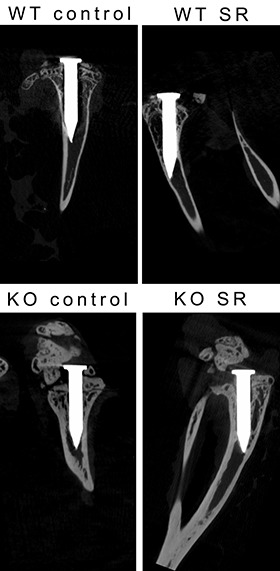
Sagittal-sectional micro-computed tomography scans of titanium implants. SR: strontium ranelate; WT: wild type; KO: knockout.

CT scanning showed clear distinctions in the microstructure of bone among the four experimental groups. In Figure 2, although some images of the surrounding bone were hidden in the shadow of the Ti pin, the greatest severity of osteolysis was observed around the pin in the control group.

**Figure 3.**
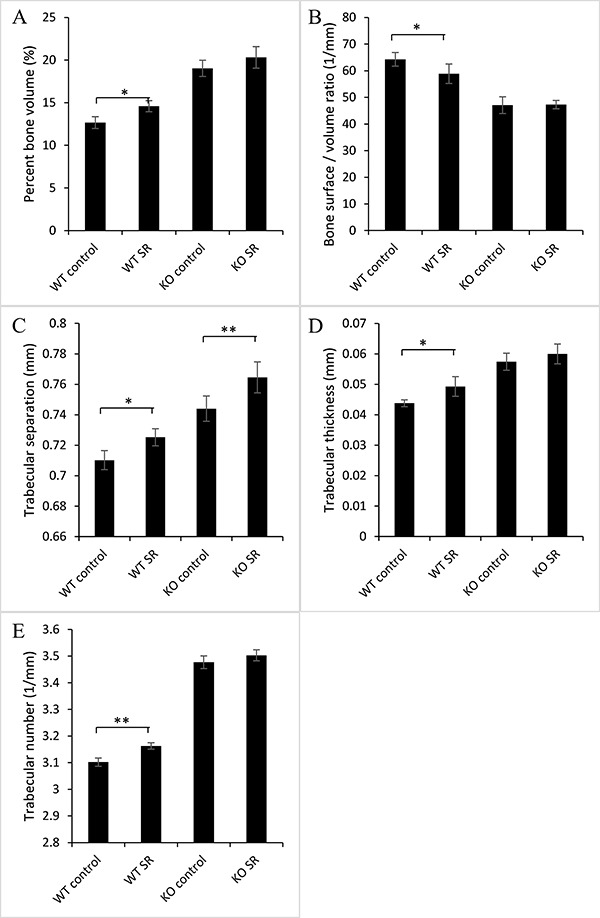
Micro-computed tomography analysis of bone microstructure. *A*, percent bone volume; *B*, bone surface/volume ratio; *C*, trabecular separation; *D*, trabecular thickness; *E*, trabecular number. Data are reported as means±SD. *P<0.05, **P<0.01 (ANOVA). SR: strontium ranelate; WT: wild type; KO: knockout.

The presence of SR increased BV/TV in the WT and KO groups (12.67±0.69% in WT control group, 14.59±0.64% in WT SR group, 19.02±0.95% in KO control group, and 20.32±1.27% in KO SR group). The results of one-way ANOVA showed that there was a significant difference between the WT control group and the WT SR group (P=0.034). There was a significant difference between the WT SR group and the KO SR group (P=0.000), and there was no statistical difference between the KO control group and the KO SR group (P=0.124). BS/BV was significantly decreased in the SR groups (64.25±2.58/mm in WT control group, 58.85±3.66/mm in WT SR group, 47.09±3.14/mm in KO control group, and 47.28±1.56/mm in KO SR group). The results of one-way ANOVA showed that there was a significant difference between the WT control group and the WT SR group (P=0.048). There was a significant difference between the WT SR group and the KO SR group (P=0.001), and there was no statistical difference between the KO control group and the KO SR group (P=0.935). In addition, TbTh was significantly increased in the SR groups (0.04±0.001 mm in WT control group, 0.05±0.003 mm in WT SR group, 0.06±0.003 mm in KO control group, and 0.06±0.003 mm in KO SR group). There was a significant difference between the WT control group and the WT SR group (P=0.041), and between the WT SR group and the KO SR group (P=0.001). There was no statistical difference between the KO control group and the KO SR group (P=0.228). TbN significantly increased in the SR groups (3.10±0.02/mm in WT control group, 3.16±0.01/mm in WT SR group, 3.48±0.02/mm in KO control group, and 3.50±0.02/mm in KO SR group). A significant difference was found between the WT control group and the WT SR group (P=0.004), and between the WT SR group and the KO SR group (P=0.000). There was no statistical difference between the KO control group and the KO SR group (P=0.129). TbSp was significantly decreased in the SR groups (0.71±0.006 mm in WT control group, 0.73±0.006 mm in WT SR group, 0.74±0.008 mm in KO control group, and 0.76±0.010 mm in KO SR group). A significant difference was found between the WT control group and the WT SR group (P=0.045), and between the WT SR group and the KO SR group (P=0.000). There was no statistical difference between the KO control group and the KO SR group (P=0.012). (Figure 3).

### SR treatment promoted a decrease of inflammatory factors in mouse sera

**Figure 4.**
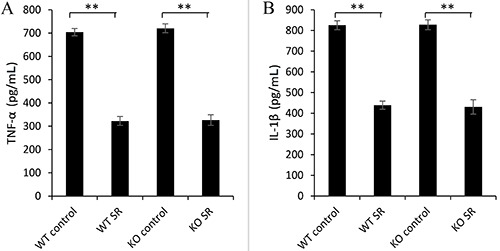
Serum levels of tumor necrosis factor (TNF)-α (*A*) and interleukin (IL)-1β (*B*) obtained by ELISA. Data are reported as means±SD. **P<0.01 (ANOVA). SR: strontium ranelate; WT: wild type; KO: knockout.

Serum ELISA showed that the levels of TNF-α in the serum of the SR group were significantly decreased (704±15.63 pg/mL in WT control group, 323±19.22 pg/mL in WT SR group, 721±18.77 pg/mL in KO control group, and 327±23.03 pg/mL in KO SR group) and there was a significant difference between the WT control group and the WT SR group (P=0.000), between the WT SR group and the KO SR group (P=0.806), and between the KO control group and the KO SR group (P=0.000). The level of IL-1β was significantly lower than in the control groups (825±21.58 pg/mL in WT control group, 439±19.67 pg/mL in WT SR group, 828±23.63 pg/mL in KO control group, and 431±34.79 pg/mL in KO SR group). The results of one-way ANOVA showed that there was a significant difference between the WT control group and the WT SR group (P=0.000), and between the KO control group and the KO SR group (P=0.000). There was no statistical difference between the WT SR group and the KO SR group (P=0.701) (Figure 4).

### Treatment of SR improved periprosthetic bone formation and osteoclastic factor synthesis

The results of western blotting showed that the levels of β-catenin, Runx2, osteocalcin (OCN), and OPG in the WT SR group were significantly higher than those in the WT control group, but there was no statistical difference between the KO control group and KO SR group.

For β-catenin, the values were 1.04±0.05 in the WT control group, 1.22±0.02 in the WT SR group, 1.78±0.02 in the KO control group, and 1.79±0.04 in the KO SR group. The results of one-way ANOVA showed that there was a significant difference between the WT control group and the WT SR group (P=0.000) and between the WT SR group and the KO SR group (P=0.000), but no statistical difference was found between the KO control group and the KO SR group (P=0.912).

For Runx2, the values were 0.34±0.005 in the WT control group, 0.47±0.010 in the WT SR group, 0.94±0.013 in the KO control group, and 0.89±0.005 in the KO SR group. One-way ANOVA showed that there was a significant difference between the WT control group and the WT SR group (P=0.000), between the WT SR group and the KO SR group (P=0.000), and between the KO control group and the KO SR group (P=0.000).

For OCN, the values were 0.72±0.007 in the WT control group, 0.98±0.010 in the WT SR group, 1.10±0.007 in the KO control group, and 1.11±0.007 in the KO SR group. One-way ANOVA showed that there was a significant difference between the WT control group and the WT SR group (P=0.000) and between the WT SR group and the KO SR group (P=0.000), but there was no statistical difference between the KO control group and the KO SR group (P=0.058).

For OPG, the values were 0.59±0.03 in the WT control group, 0.90±0.02 in the WT SR group, 1.32±0.04 in the KO control group, and 1.32±0.04 in the KO SR group. One-way ANOVA showed that there was a significant difference between the WT control group and the WT SR group (P=0.000) and between the WT SR group and the KO SR group (P=0.000), but there was no statistical difference between the KO control group and the KO SR group (P=0.873).

SR decreased the levels of SOST and RANKL, but there was no significant difference between the KO control group and the KO SR group.

For SOST, the values were 1.26±0.03 in the WT control group and 0.51±0.01 in the WT SR group. The results of Student's *t*-test showed that there was a significant difference between the WT control group and the WT SR group (P=0.000).

**Figure 5.**
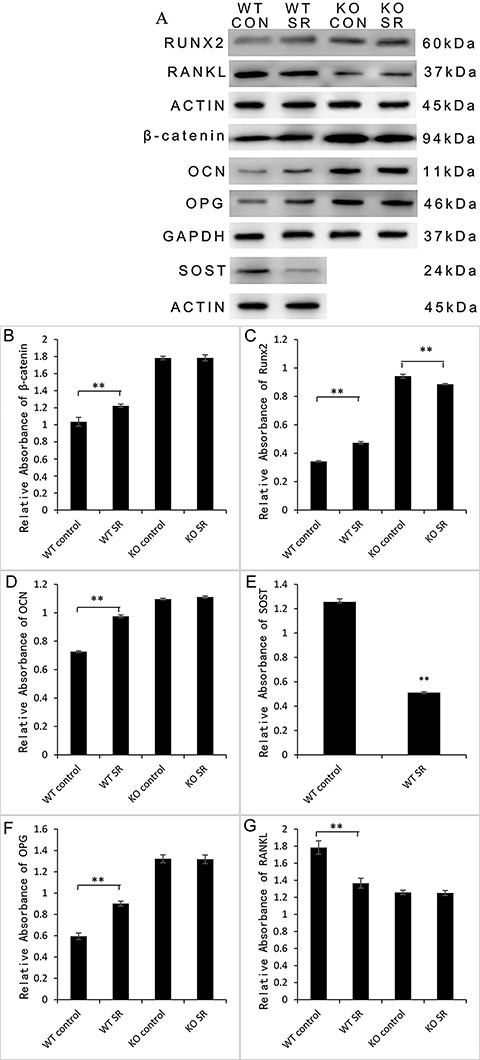
Western blot analysis of proteins in bone tissue surrounding the implant. The results are reported as the ratio of the target protein to the internal control (β-actin). The data are reported as means±SD of three experiments. *A*: western blot; *B*: β-catenin; *C*: runx2; *D*: osteocalcin (OCN); *E*: sclerostin (SOST); *F*: osteoprotegerin (OPG); *G*: receptor activator for nuclear factor-κB ligand (RANKL). **P<0.01 (ANOVA). SR: strontium ranelate; WT: wild type; KO: knockout.

For RANKL, the values were 1.78±0.08 in the WT control group, 1.37±0.06 in the WT SR group, 1.26±0.03 in the KO control group, and 1.25±0.03 in the KO SR group. The results of one-way ANOVA showed that there was a significant difference between the WT control group and the WT SR group (P=0.000) and between the WT SR group and the KO SR group (P=0.028), but there was no statistical difference between the KO control group and the KO SR group (P=0.879) (Figure 5).

## Discussion

Previous studies have shown that the pathogenesis of aseptic loosening is complex and the result of a combination of multiple factors. An imbalance in bone formation and osteolysis around the prosthesis is critical to the development of aseptic loosening (2,24).

SR is an effective drug for treating osteoporosis, which can promote the activity of osteoblasts while inhibiting the formation and differentiation of osteoclasts, indicating its potential applications in the treatment of aseptic loosening (20,21). Our results showed that SR down-regulated the level of SOST, promoted bone formation, and inhibited osteolysis, resulting in the inhibition of aseptic loosening in an aseptic loosening model of female mice. This is the first study to show that SR inhibited titanium particles caused by aseptic loosening, mainly by down-regulating SOST levels.

Previous experiments have shown that SR inhibits the release of SOST from osteoblasts and promotes the differentiation and proliferation of osteoblasts by relieving the inhibitory effect of SOST on the canonical Wnt pathway (25). OPG is a downstream product of the canonical Wnt pathway and is crucial for the differentiation and proliferation of osteoclasts. Some researchers demonstrated that SR inhibits the proliferation and differentiation of osteoclasts by up-regulating the ratio of OPG/RANKL (26). In this study, we also found that SR down-regulated the levels of SOST and RANKL in the surrounding tissues of the prostheses and upregulated the levels of β-catenin, OPG, and OCN, indicating that SR inhibited aseptic loosening caused by abrasive particles by down-regulating the level of SOST and releasing the inhibition of the classical Wnt pathway activity, thereby promoting bone formation and inhibiting bone resorption. Choudhary et al. (27) showed that SR can also inhibit the activation of osteoclasts by downregulating the levels of inflammatory factors such as TNF-α and IL-1β in the serum, reducing bone dissolution around the wear particles, which is consistent with our experimental results.

µCT scanning showed that the quality of bone in the SR group was better than that in the control group. The BV/TV, BS/BV, TbTh, and TbN in the SR group and control group were significantly different, and these results were consistent with previous studies (26,28). However, the sagittal and cross-sectional scans showed that the gap between the prosthesis and surrounding tissue in the SR group was significantly smaller than that in the control group, indicating that SR improved the quality of bone in tissues surrounding the prostheses. By studying the osteolytic model in wild-type mice, we found that SR had a therapeutic effect on aseptic loosening induced by titanium particles. Our results were consistent with the results obtained from previous experiments (26,29).

Examination of SOST knockout mice revealed that the parameters of the bone around the prosthesis between the KO SR group and KO control group were essentially the same according to CT scans. Western blotting of OCN revealed no significant differences between the two groups.

We found that serum levels of TNF-α, IL-1β, and PGE2 in the KO SR group were lower than those in the KO control group, but no significant difference was observed in the ratio of OPG to RANKL between groups. This indicates that SR inhibits the inflammatory response caused by titanium particles, but does not significantly affect the balance between bone formation and osteolysis without involvement of the SOST protein. After knocking out the SOST gene, SR showed no effect on osteolysis around the titanium particles. Thus, SOST may be a key factor in the regulation of bone abnormalities around the prosthesis induced by titanium particles.

In summary, SR inhibited osteolysis from the titanium particles around the prosthesis in a female mouse aseptic loosening model, and this inhibition mainly depended on down-regulation of SOST levels. However, previous studies reported that SR has serious side effects and safety concerns in practical applications (30–32). Given its excellent application value, it is necessary to explore possible solutions for overcoming these limitations. Previous studies showed that topical application of SR is a promising method (33), while another experiment demonstrated that coating the prosthesis with strontium inhibited the aseptic loosening effect (34,35).

## References

[1] Shin DK, Kim MH, Lee SH, Kim TH, Kim SY (2012). Inhibitory effects of luteolin on titanium particle-induced osteolysis in a mouse model. Acta Biomater.

[2] Yang H, Xu Y, Zhu M, Gu Y, Zhang W, Shao H (2016). Inhibition of titanium-particle-induced inflammatory osteolysis after local administration of dopamine and suppression of osteoclastogenesis via d2-like receptor signaling pathway. Biomaterials.

[3] Liu S, Virdi AS, Sena K, Sumner DR (2012). Sclerostin antibody prevents particle-induced implant loosening by stimulating bone formation and inhibiting bone resorption in a rat model. Arthritis Rheum.

[4] Albers J, Keller J, Baranowsky A, Beil FT, Catala-Lehnen P, Schulze J (2013). Canonical wnt signaling inhibits osteoclastogenesis independent of osteoprotegerin. J. Cell Biol.

[5] Chen B, Li XD, Liu DX, Wang H, Xie P, Liu ZY (2012). Canonical wnt signaling is required for panax notoginseng saponin-mediated attenuation of the rankl/opg ratio in bone marrow stromal cells during osteogenic differentiation. Phytomedicine.

[6] Rybchyn MS, Slater M, Conigrave AD, Mason RS (2011). An akt-dependent increase in canonical wnt signaling and a decrease in sclerostin protein levels are involved in strontium ranelate-induced osteogenic effects in human osteoblasts. J Biol Chem.

[7] Chang MK, Kramer I, Keller H, Gooi JH, Collett C, Jenkins D (2014). Reversing lrp5-dependent osteoporosis and sost deficiency-induced sclerosing bone disorders by altering wnt signaling activity. J Bone Miner Res.

[8] Kedlaya R, Veera S, Horan DJ, Moss RE, Ayturk UM, Jacobsen CM (2013). Sclerostin inhibition reverses skeletal fragility in an lrp5-deficient mouse model of oppg syndrome. Sci Transl Med.

[9] Ndip A, Williams A, Jude EB, Serracino-Inglott F, Richardson S, Smyth JV (2011). The rankl/rank/opg signaling pathway mediates medial arterial calcification in diabetic charcot neuroarthropathy. Diabetes.

[B10] Theoleyre S, Wittrant Y, Tat SK, Fortun Y, Redini F, Heymann D (2004). The molecular triad opg/rank/rankl: involvement in the orchestration of pathophysiological bone remodeling. Cytokine Growth Factor Rev.

[11] Ferreira E, Bortolin RH, Freire FP, Souza K, Bezerra JF, Ururahy M (2017). Zinc supplementation reduces rankl/opg ratio and prevents bone architecture alterations in ovariectomized and type 1 diabetic rats. Nutr. Res.

[12] Li X, Zhang Y, Kang H, Liu W, Liu P, Zhang J (2005). Sclerostin binds to lrp5/6 and antagonizes canonical wnt signaling. J Biol Chem.

[13] van Bezooijen RL, Roelen BA, Visser A, van der Wee-Pals L, de Wilt E, Karperien M (2004). Sclerostin is an osteocyte-expressed negative regulator of bone formation, but not a classical bmp antagonist. J Exp Med.

[14] Suen PK, Zhu TY, Chow DH, Huang L, Zheng LZ, Qin L (2015). Sclerostin antibody treatment increases bone formation, bone mass, and bone strength of intact bones in adult male rats. Sci Rep.

[15] Pioletti DP, Kottelat A (2004). The influence of wear particles in the expression of osteoclastogenesis factors by osteoblasts. Biomaterials.

[16] Shao H, Shen J, Wang M, Cui J, Wang Y, Zhu S (2015). Icariin protects against titanium particle-induced osteolysis and inflammatory response in a mouse calvarial model. Biomaterials.

[17] Lam J, Takeshita S, Barker JE, Kanagawa O, Ross FP, Teitelbaum SL (2000). Tnf-alpha induces osteoclastogenesis by direct stimulation of macrophages exposed to permissive levels of rank ligand. J Clin Invest.

[18] Wang H, Jia T, Zacharias N, Gong W, Du H, Wooley PH (2012). Combination gene therapy targeting on interleukin-1β and rankl for wear debris-induced aseptic loosening. Gene Ther.

[19] Stea S, Visentin M, Granchi D, Melchiorri C, Soldati S, Sudanese A (1999). Wear debris and cytokine production in the interface membrane of loosened prostheses. J Biomater Sci Polym Ed.

[20] Reginster JY, Brandi ML, Cannata-Andia J, Cooper C, Cortet B, Feron JM (2015). The position of strontium ranelate in today's management of osteoporosis. Osteoporos Int.

[21] Karakan NC, Akpinar A, Goze F, Poyraz O (2017). Investigating the effects of systemically administered strontium ranelate on alveolar bone loss histomorphometrically and histopathologically on experimental periodontitis in rats. J Periodontol.

[22] Yang S, Yu H, Gong W, Wu B, Mayton L, Costello R (2007). Murine model of prosthesis failure for the long-term study of aseptic loosening. J Orthop Res.

[23] Sun S, Guo H, Zhang J, Yu B, Sun K, Jin Q (2013). Adenovirus-mediated expression of bone morphogenetic protein-2 activates titanium particle-induced osteoclastogenesis and this effect occurs in spite of the suppression of tnf-alpha expression by sirna. Int J Mol Med.

[24] Xie J, Hou Y, Fu N, Cai X, Li G, Peng Q (2015). Regulation of extracellular matrix remodeling proteins by osteoblasts in titanium nanoparticle-induced aseptic loosening model. J Biomed Nanotechnol.

[25] Bao X, Liu X, Zhang Y, Cui Y, Yao J, Hu M (2014). Strontium promotes cementoblasts differentiation through inhibiting sclerostin expression *in vitro*. Biomed Res Int.

[26] Liu X, Zhu S, Cui J, Shao H, Zhang W, Yang H (2014). Strontium ranelate inhibits titanium-particle-induced osteolysis by restraining inflammatory osteoclastogenesis in vivo. Acta Biomater.

[27] Choudhary S, Halbout P, Alander C, Raisz L, Pilbeam C (2007). Strontium ranelate promotes osteoblastic differentiation and mineralization of murine bone marrow stromal cells: involvement of prostaglandins. J Bone Miner Res.

[28] Lu YC, Chang TK, Yeh ST, Fang HW, Lin CY (2015). The potential role of strontium ranelate in treating particle-induced osteolysis. Acta Biomater.

[29] Li Y, Li X, Song G, Chen K, Yin G, Hu J (2012). Effects of strontium ranelate on osseointegration of titanium implant in osteoporotic rats. Clin Oral Implants Res.

[30] Rossini M, Adami G, Adami S, Viapiana O, Gatti D (2016). Safety issues and adverse reactions with osteoporosis management. Expert Opin Drug Saf.

[B31] Lee HY, Shen MX, Lim YL, Tay YK, Chan MM, Pang SM (2016). Increased risk of strontium ranelate-related sjs/ten is associated with hla. Osteoporos Int.

[32] Geng T, Sun S, Chen X, Wang B, Guo H, Zhang S (2018). Strontium ranelate reduces the progression of titanium particle-induced osteolysis by increasing the ratio of osteoprotegerin to receptor activator of nuclear factor-kappab ligand in vivo. Mol Med Rep.

[33] Guo X, Wei S, Lu M, Shao Z, Lu J, Xia L (2016). Dose-dependent effects of strontium ranelate on ovariectomy rat bone marrow mesenchymal stem cells and human umbilical vein endothelial cells. Int J Biol Sci.

[34] Gu Z, Huang B, Li Y, Tian M, Li L, Yu X (2016). Strontium-doped calcium polyphosphate/ultrahigh molecular weight polyethylene composites: a new class of artificial joint components with enhanced biological efficacy to aseptic loosening. Mater Sci Eng C Mater Biol Appl.

[35] Tian A, Zhai JJ, Peng Y, Zhang L, Teng MH, Liao J (2014). Osteoblast response to titanium surfaces coated with strontium ranelate-loaded chitosan film. Int J Oral Maxillofac Implants.

